# *ARGOS* Genes in Cauliflower: Genome-Wide Identification and Functional Validation of *BobARL2* Under Abiotic Stresses

**DOI:** 10.3390/ijms26199810

**Published:** 2025-10-09

**Authors:** Mengmeng Duan, Guixiang Wang, Mei Zong, Shuo Han, Ning Guo, Fan Liu

**Affiliations:** State Key Laboratory of Vegetable Biobreeding, National Engineering Research Center for Vegetables, Beijing Key Laboratory of Vegetable Germplasm Improvement, Key Laboratory of Biology and Genetic Improvement of Horticultural Crops (North China), Beijing Vegetable Research Center, Beijing Academy of Agriculture and Forestry Sciences, Beijing 100097, China

**Keywords:** Cauliflower, Auxin-Regulated Gene Involved in Organ Size (ARGOS), abiotic stress, *BobARL2*

## Abstract

The Auxin-Regulated Gene Involved in Organ Size (ARGOS) proteins have crucial regulatory effects on organ size and responses to environmental stresses. Despite their importance, *Brassica oleracea* ARGOS gene members and their functions in response to abiotic stresses have not been thoroughly investigated. In this study, we identified 40 ARGOS genes via a genome wide analysis of cauliflower and two other *B. oleracea* morphotypes as well as *Brassica rapa*, *Brassica nigra*, and *Raphanus sativus*. Expression pattern analyses indicated that these genes are responsive to multiple abiotic stresses, including salinity, heat, cold, and diverse hormones. Notably, the expression of an *ARGOS-like* gene (*BobARL2*) was upregulated in cauliflower treated with 1-aminocyclopropane-1-carboxylic acid (ACC). Moreover, the overexpression of *BobARL2* decreased ethylene sensitivity, resulting in less inhibition of root elongation compared to the wild-type. Additionally, the overexpression lines exhibited enhanced salt tolerance. A yeast two-hybrid assay and luciferase complementation imaging (LCI) assay confirmed that BobARL2 can interact with Reversion-to-ethylene sensitivity Like4 (BobRTL4), which negatively regulates ethylene signal transduction. These findings advance our understanding of the evolution and functional roles of ARGOS genes in cauliflower and other Brassicaceae species, particularly in relation to abiotic stress responses, while also offering valuable insights relevant to the genetic improvement and breeding of novel varieties.

## 1. Introduction

*Brassica oleracea* (2n = 2x = 18, CC), a species within the Brassicaceae family, is one of the three diploid species in the triangle of U, along with *B*. *rapa* (AA) and *B*. *nigra* (BB). This species includes a variety of economically important vegetable crops that display remarkable morphological diversity. This is exemplified by the leafy head of cabbage, the tuberous stem of kohlrabi, the succulent stem of Chinese kale, the axillary heading buds of Brussels sprouts, and the enlarged, arrested inflorescences (curds) of cauliflower and broccoli [1]. The development of these organs can be influenced by abiotic stresses, such as drought and cold, as well as by hormonal changes [2,3,4]. Despite significant advancements, domestication processes and the mechanisms responsible for the phenotypic diversity in these distinct organs remain largely unclear, thereby necessitating continued research on the development of specific organs. Although population resequencing studies have identified several genomic selection signals associated with specific morphotypes [1,5,6,7], the genetic mechanisms underlying this rapid evolution and domestication remain relatively uncharacterized. Some studies suggest that the genetic diversity in Brassica species may be linked to hormonal responses and environmental adaptability [1,7,8,9]; however, the specific genes mediating responses to environmental conditions and the underlying mechanisms require further investigation. Identifying genes related to environmental responses and organ development is crucial for further elucidating the development of Brassica vegetable organs and for advancing crop improvement.

*ARGOS* gene members encode a class of hormone-responsive proteins that are prevalent across various plant species. These proteins play a crucial role in regulating organ size and responding to environmental stresses [10,11,12,13,14]. ARGOS proteins are characterized by a conserved organ size-related (OSR) domain containing a distinct LPPLPPPP motif and two transmembrane helices, both of which are essential for promoting organ growth [10,11,12,13,14,15]. These proteins are primarily localized in membranes (e.g., endoplasmic reticulum, Golgi apparatus, and cell) [16]. In Arabidopsis, four *ARGOS* genes have been identified: *AtARGOS*, *AtARL*, *Organ size related 1* and *2* (*AtOSR1* and *AtOSR2*). Orthologs have been characterized in several crop species, with five, eight, and four genes detected in *Oryza sativa* [17], *Zea mays* [18], and *Nicotiana tabacum*, respectively [19]. These genes encode proteins with distinct functions during the development of various organs and different stress conditions. The expression of *ARGOS* is induced by auxin and cytokinin in both Arabidopsis and rice. Ectopic expression prolongs the expression of *Aintegumenta* (*ANT*) and *Cyclin D3;1* (*CycD3;1*), thereby enhancing cell division and promoting lateral organ growth [15,17,20]. Separately, in poplar, the auxin transporter Walls Are Thin1 (WAT1) has been shown to interact with PagARGOS to regulate xylem development [21]. Similarly, *ARL* expression is induced by brassinosteroids via the receptor BR INSENSITIVE 1 (BRI1) in Arabidopsis and rice, leading to increased cell enlargement due to cell volume regulation [17,19]. By contrast, the expression of *OSR1* and *OSR2* was downregulated by abscisic acid and brassinosteroids in Arabidopsis [10,11]. Ethylene upregulates *ARL* expression, further promoting cell expansion in Arabidopsis and tobacco [19,22]. Ethylene also upregulates *OSR1* and *OSR2* in Arabidopsis, as well as *ARGOS* and *OSR1* in maize [10,12,13,14]. Notably, several ARGOS proteins are negative regulators of ethylene signaling. Earlier research revealed the similarity in the localization of ARGOS1 and ethylene receptors [16]. In maize, ZmARGOS8 and ZmOSR1 interact with Reversion-to-ethylene sensitivity1 (ZmRTE1), ZmRTL4, and ZmRTL2 to attenuate ethylene signaling [13]. There is also research showing that *ARGOS* genes are also involved in stress response. For example, *ARGOS* expression is upregulated by drought and salt stress; the overexpression of *ARGOS* enhances drought and salt resistance in wheat [23]. Similarly, overexpression of ZmARGOS8 in maize decreases ethylene sensitivity and improves yield under stress conditions [12,13,14]. Moreover, *TaARGOS-D* expression in common wheat is upregulated under drought and salinity stress conditions; its overexpression enhances Arabidopsis stress tolerance [23]. These findings provide valuable insights for elucidating the functions of *ARGOS* genes during stress responses.

Cauliflower is a vegetable crop that is cultivated and consumed worldwide. The curd is composed of fleshy and enlarged stems and developmentally arrested inflorescence meristems. Cauliflower reproductive development is highly susceptible to environmental conditions, making organ expansion and stress resistance crucial in influencing yield and quality. Previous studies showed that *ARGOS* genes are essential for organ enlargement and stress resistance. Although recent genomic studies of Brassicaceae species have enhanced our understanding of their gene families [6,24,25,26,27,28], *ARGOS* genes in *B. oleracea* have not been comprehensively investigated. Hence, in this study, the *ARGOS* genes in cauliflower and two other *B. oleracea* morphotypes were thoroughly analyzed and compared with the corresponding family in other Brassicaceae species, including *B. rapa*, *B. nigra*, and *R*. *sativus.* We characterized these *ARGOS* genes in terms of their phylogenetic relationships, exon–intron organization, conserved motifs, and promoter cis-acting elements. Notably, *BobARL2* was revealed to be highly expressed in cauliflower leaf, root, and curd. Furthermore, the expression of *BobARGOS* genes was induced by various abiotic stresses (e.g., ethylene and NaCl treatment). These findings, combined with experimental validation of the interaction between BobARL2 and BobRTL4, suggest that BobARL2 may enhance abiotic stress tolerance by interacting with BobRTL4 to decrease ethylene sensitivity. This study provides a foundation for future studies aimed at elucidating the molecular mechanisms through which BobARL2 mediates hormone and stress responses in cauliflower.

## 2. Results

### 2.1. Genome-Wide Identification and Characterization of ARGOS Genes

On the basis of sequence homology and conserved domains, seven, seven, and five *ARGOS* genes were identified in three *B. oleracea* accessions: kale-like rapid cycling morphotype (TO1000, Bo) [24], broccoli (HDEM, Boi) [29], and cauliflower (Korso, Bob) [6], respectively. Additionally, six, eight, and seven *ARGOS* genes were identified in Chinese cabbage (Chiifu, Bra) [27], black mustard (YZ12151, Bni) [28], and radish (XYB-36-2, Rs) [26], respectively. These genes were named according to their homology with *ARGOS* genes in Arabidopsis (Table S1). Proteins encoded by these genes comprised 59 (BoOSR2.1) to 491 (BoARGOS2) amino acids, with molecular weights between 6.47 kDa (BoOSR2.1) and 57.68 kDa (BoARGOS2) and isoelectric points ranging from 3.34 (BniOSR2.1) to 11.57 (BniARGOS1) (Table S1). BraOSR2.2, BoiOSR2.2, BniOSR2.2, and RsARL3 contained a single transmembrane domain, whereas BobARL1 had three transmembrane domains. The other 35 ARGOS proteins from various species included two transmembrane domains, underscoring the evolutionary conservation of these genes. All identified ARGOS proteins share a conserved C-terminal OSR domain that is sufficient to promote organ growth, a feature consistent with ARGOS proteins from diverse species (Figure S1). Moreover, comparative analysis revealed that OSR1 and OSR2 proteins across different species contain incomplete OSR domains, in contrast to the full-length domains found in ARGOS and ARL.

**Figure 1:**
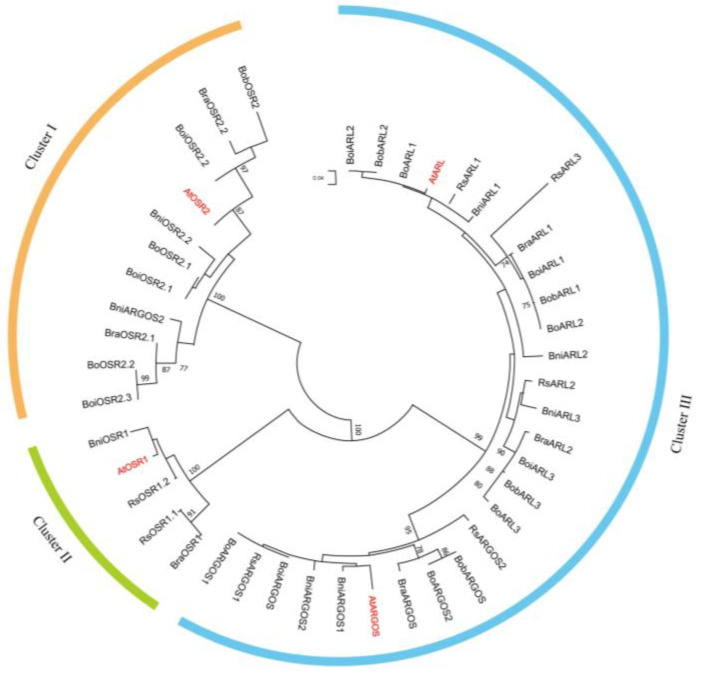
Evolutionary relationships among ARGOS proteins from Arabidopsis, *B. oleracea* (kale-like rapid cycling, cauliflower and broccoli), *B. rapa*, *R. sativus* and *B. nigra*. The phylogenetic tree was constructed employing the Neighbor-Joining algorithm in MEGA-7.0, and the reliability of the branches was tested (1000 bootstrap replicates). The ARGOS proteins are classified into three major clades (Cluster I-III). Proteins in Group I-III are highlighted in orange, green and blue lines, respectively.

To investigate the similarity and evolutionary history of *ARGOS* genes in Brassicaceae species, we performed a phylogenetic analysis using ARGOS protein sequences from Arabidopsis, kale-like rapid cycling morphotype, broccoli, cauliflower, Chinese cabbage, black mustard, and radish (Figure 1, Table S2). Using Arabidopsis as a reference, 40 *ARGOS* genes from six species were classified into three clusters (Clusters I–III). Cluster I included *AtOSR2* (AT2G41225) and its homologs in kale-like rapid cycling morphotypes, broccoli, cauliflower, black mustard, radish, and Chinese cabbage. Similarly, Cluster II contained *AtOSR1* (AT2G41230) and its homologs in the related species. Notably, *AtOSR1* homologs were absent in all three *B. oleracea* accessions. Synteny analysis confirmed that the entire genomic region harboring *OSR1* in Arabidopsis is missing in *B. oleracea* (Table S3), indicating that the loss of this gene resulted from a species-specific deletion event during genome duplication and fragmentation. Cluster III comprised *AtARGOS* and *AtARL* along with their homologs; the sequences of these genes were highly similar across species.

### 2.2. Structural Analysis of ARGOS Genes 

**Figure 2:**
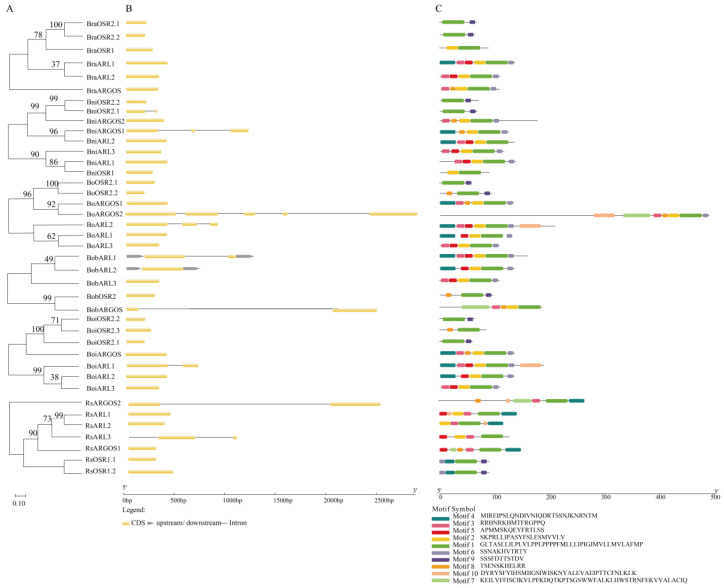
Unrooted neighbor-joining phylogenetic tree, conserved protein motifs, and structural analysis of *ARGOS* genes. (**A**) Evolutionary relationships of the ARGOS proteins in six species. (**B**) The structures of the 40 putative *ARGOS* genes. The UTRs, exons, and introns are represented by grey boxes, yellow boxes, and black lines, respectively. (**C**) Conserved motif analysis of the ARGOS proteins. The different motifs are indicated by different colored boxes numbered from motif 1 to motif 10.

We further analyzed the phylogenetic relationships of different paralogs of *ARGOS* genes in six distinct accessions of *B. oleracea*, *B. rapa*, *B. nigra*, and *R. sativus* to clarify the diversity and similarity of *ARGOS* genes within species (Figure 2A). A structural analysis revealed that the number of exons in *ARGOS* genes varied from one to five across species, reflecting species-specific characteristics (Figure 2B). Notably, all *ARGOS* genes in Chinese cabbage lacked introns. Homologs across species exhibited common features. For example, with the exception of *BniOSR2.1*, *OSR* genes in other species lacked introns. We subsequently screened 40 *ARGOS* homologs in the six selected accessions and identified 10 motifs (Figure 2C). All *ARGOS* homologs contained Motif 1, which corresponds to the conserved OSR domain (i.e., gene family characteristic). Almost all *OSR2* homologs contained both Motifs 1 and 9 (the exception was *BoiOSR2.3*). Interestingly, *OSR* genes had significantly fewer motifs than the other *ARGOS* genes. Additionally, except for *RsARL2*, *ARL* genes in different species consistently contained Motifs 2 and 5, whereas most *ARGOS* genes lacked Motif 5 (Figure 2C). The distinct motif compositions among subclades suggest potential structural bases for functional diversification within the *ARGOS* family. These conserved and divergent patterns provide valuable clues for future investigations into the specific roles of different motifs.

### 2.3. Promoter Cis-Acting Elements in ARGOS Genes

**Figure 3:**
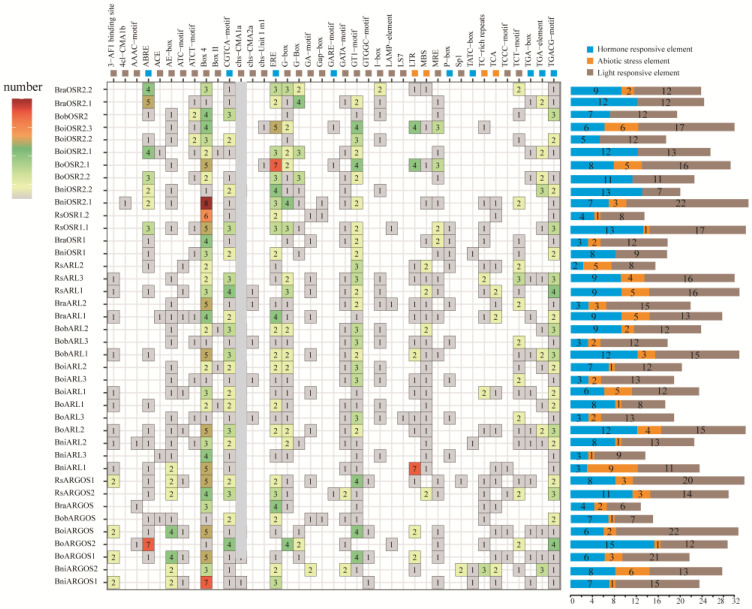
Cis-element analysis of promoters of *ARGOS* genes. The 2000 bp promoter regions were analyzed, and the number of predicted elements is represented by the color scale. The multicolor histogram displays the number of various cis-regulatory element categories across these genes.

An analysis of *ARGOS* gene promoters identified 39 major cis-acting elements (Figure 3), which were broadly classified into the following three main categories: light-responsive, hormone-responsive, and stress-responsive cis-acting elements. This indicates that the *ARGOS* genes in Brassicaceae may be predominantly regulated by these three factors. Light-responsive elements were the most abundant, accounting for more than 50% of all identified cis-acting elements. These elements included AE-box, 3-AF1 binding site, GT1-motif, AAAC-motif, 4cl-CMA1b, Sp1, and Box 4, among others, underscoring the critical regulatory effect of light on *ARGOS* gene expression. The identified hormone-responsive elements included ABRE (abscisic acid response), TGA-box and TGA-element (auxin response), ERE (ethylene response), TATC-box, P-box, and GARE-motif (gibberellin response), CGTCA-motif and TGACG-motif (methyl jasmonate response), O2-site (cytokinin response), and TCA-element (salicylic acid response). Hence, *ARGOS* genes are regulated by various hormone signaling pathways. Additionally, several stress-responsive elements were identified, including TC-rich repeats and MBS (drought stress response) and LTR (low-temperature response).

The number and types of cis-acting elements varied significantly among species and varieties. Specifically, *BniOSR2.1* had the most cis-acting elements (32), compared to only 13 in both *RsOSR1.2* and *BniARL3*. Notably, the light-responsive element Box-4 was detected in all *ARGOS* genes. The 3-AF1 binding site motif was not detected in any of the *OSR* genes across all analyzed species. Moreover, the methyl jasmonate-responsive elements (TGSCG-motif) were present in most genes (exceptions were *OSR2.1* in kale-like rapid cycling morphotype, and *OSR2.3* in broccoli, *BraARGOS* in Chinese cabbage and *RsARL2* in radish). In addition, the light-responsive element chs-Unit 1 ml was identified only in kale-like rapid cycling morphotype and broccoli, while the LS7 element was exclusive to kale-like rapid cycling morphotype. Furthermore, the cytokinin-responsive element TATC-box was present only in *B. nigra ARGOS* genes.

These findings may reflect a complex regulatory network in which *ARGOS* genes affect various hormone signaling and stress response pathways. The observed differences in the number and types of cis-acting elements among *ARGOS* members may indicate functional diversification, which may have contributed to their distinct roles in plant development and stress adaptation. This analysis is significant as it provides crucial insights into the mechanisms regulating the *ARGOS* genes, thereby providing the basis for future research aimed at elucidating the roles of these genes in plant growth and responses to environmental conditions.

### 2.4. BobARGOS Endoplasmic Reticulum Localization

**Figure 4:**
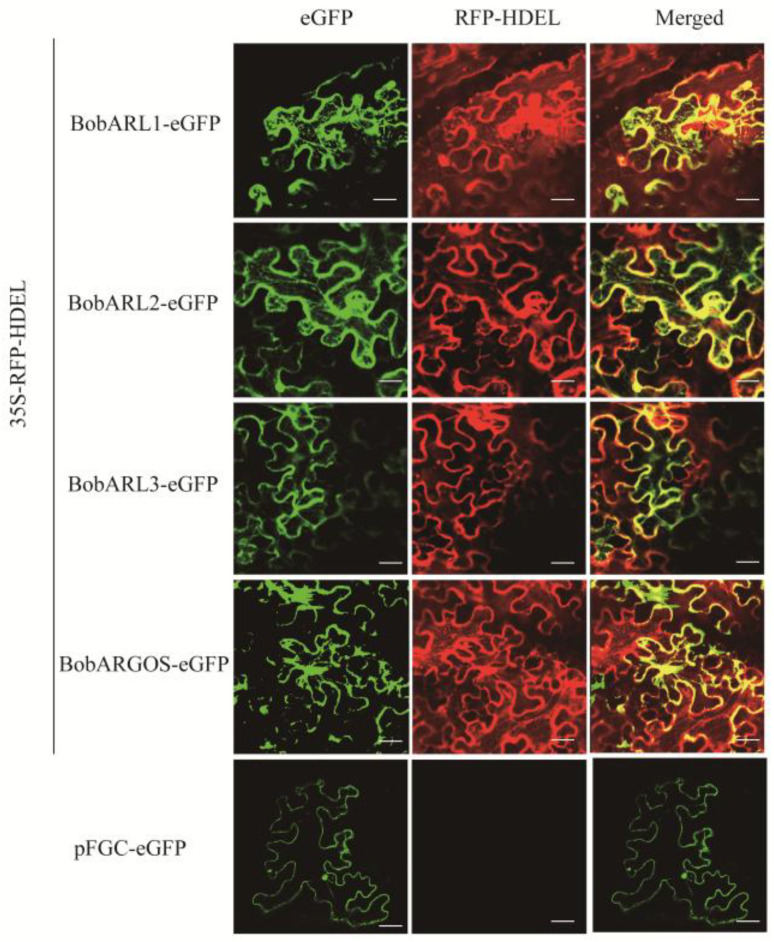
Subcellular localization of BobARGOS proteins in *N. benthamiana* epidermal cells. Confocal microscopy images showing the fluorescence signals of BobARGOS-eGFP fusion proteins (green) and the endoplasmic reticulum marker RFP-HDEL (red). The rightmost panels show the merged images, with yellow indicating co-localization. Epidermal cells expressing the empty pFGC-eGFP vector were used as a control. Scale bars = 50 µm.

According to study predictions (Section 2.1), BobARGOS proteins are most likely localized to the endoplasmic reticulum (ER). To validate these predictions, we selected several *BobARGOS*s (*BobARGOS*, *BobARL1*, *BobARL2*, and *BobARL3*) with expression levels that were significantly affected by stress conditions and constructed pFGC-BobARGOS-GFP expression vectors. Transient transformation experiments were performed via the *Agrobacterium tumefaciens*-mediated transformation of the lower epidermal cells of *N. benthamiana* leaves. On the basis of the detected fluorescence, BobARGOS, BobARL1, BobARL2, and BobARL3 were localized to the ER. These results are consistent with the predictions, suggesting that BobARGOS, BobARL1, BobARL2, and BobARL3 may have similar functions (Figure 4).

### 2.5. ARGOS Gene Expression in Various Cauliflower Organs

**Figure 5:**
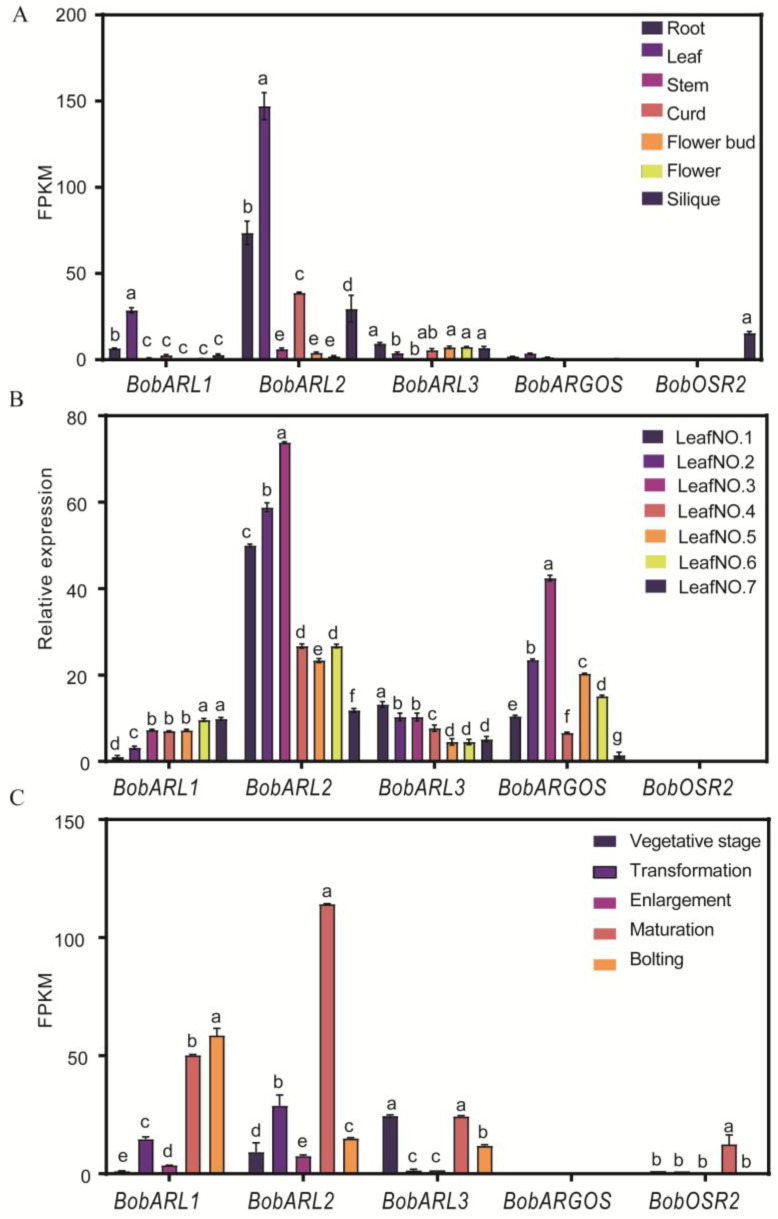
Expression analysis of *ARGOS* genes in various cauliflower organs (**A**), leaves at varying maturity stages (**B**), and curd at different development stages (**C**). Significant differences were analyzed using ordinary one-way ANOVA by Tukey’s test; lowercase letters indicate statistical significance (*p* < 0.05).

To clarify the biological functions of five *BobARGOS* genes in different cauliflower organs, we used available transcriptome data [27] to analyze their expression in various tissues (root, stem, leaf, curd, bud, flower, and silique) and different curd development stages. We also performed a quantitative real-time polymerase chain reaction (qRT-PCR) analysis to compare their expression patterns in different leaf positions (L1–L7). *BobARL1* and *BobARL2* were highly expressed in leaves, while *BobARL2* was also expressed at relatively high levels in roots and curds (Figure 5A). An analysis of expression in different leaf positions (L1–L7) showed that *BobARL1* was highly expressed in young leaves. *BobARL2* and *BobARGOS* had similar expression trends, with expression peaking in the third leaf from the outside and generally lower expression levels in younger leaves than in mature leaves. *BobARL3* was most highly expressed in mature leaves, whereas *BobOSR2* expression was almost undetectable in leaves (Figure 5B). Moreover, during curd development, *BobARL2* was significantly expressed at maturity, while *BobARL1* was expressed at high levels at maturity and during bolting. By contrast, *BobARL3* was significantly expressed during the vegetative growth stage and at maturity, while *BobARGOS* expression was almost undetectable during curd development (Figure 5C). *BobOSR2* was expressed at low levels across all examined tissues, suggesting that it may play a limited role in cauliflower under normal growth conditions.

### 2.6. Cauliflower ARGOS Gene Expression in Response to Different Abiotic Stresses

**Figure 6:**
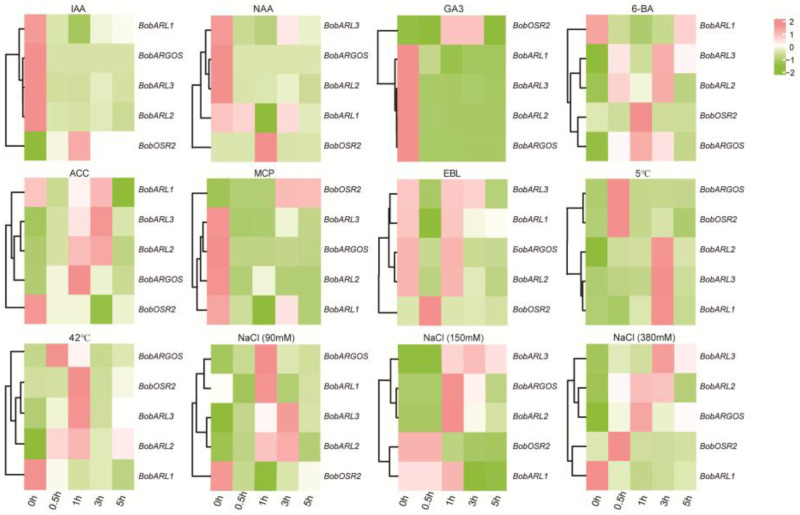
Expression analysis of *ARGOS* genes under various treatments. qRT-PCR was performed to assess transcript levels in response to abiotic stresses (cold, heat, salinity) and phytohormones (IAA, NAA, 6-BA, GA3, MCP, ACC, EBL).

Considering multiple hormone- and stress-responsive elements were identified in *BobARGOS* promoters, we analyzed the effects of different hormones and abiotic stresses on *BobARGOS* expression. More specifically, the expression levels of five *BobARGOS* genes following exposures to different stresses, including indole-3-acetic acid (IAA), 1-naphthaleneacetic acid (NAA), 6-benzylaminopurine (6-BA), 1-methylcyclopropene (1-MCP), gibberellin (GA3), ACC, 24-epibrassinolide (EBL), salinity (NaCl), and high/low temperatures, were analyzed via qRT-PCR (Figure 6). After IAA, NAA, and GA3 treatments, *BobARL2* and *BobARGOS* expression levels were significantly downregulated. This implies that *BobARL2* and *BobARGOS* may encode proteins with important roles when auxin or gibberellin levels are relatively low. The expression levels of four *BobARGOS* genes (excluding *BobOSR2*) decreased to varying degrees following the 1-MCP treatment. By contrast, *BobARL2* and *BobARL3* expression levels were significantly upregulated after the ACC treatment, indicating that these are responsive to ethylene. Furthermore, *BobARL2* and *BobARL3* expression patterns were similar in response to both 6-BA and ACC treatments, suggesting that they may play analogous roles in hormone-responsive processes. Under 6-BA and ACC treatment conditions, *BobARL1* expression was initially downregulated, but was subsequently upregulated. After the EBL treatment, *BobARL2*, *BobARL3*, and *BobARGOS* expression levels initially decreased and then increased before decreasing again. Under both cold and heat stress conditions, *BobARL2* and *BobARL3* had similar expression patterns, with transcript levels generally increasing in both cases. Following an exposure to cold stress, expression peaked at 3 h, whereas under heat stress conditions, expression peaked at 1 h and then decreased. Under NaCl stress conditions, *BobARL1* expression initially decreased, but this was followed by a transient upregulation (90 mM), biphasic response (peak at 1 h; 150 mM), or sustained downregulation (380 mM). By contrast, *BobARL2* and *BobARL3* expression levels were transiently upregulated by all NaCl treatments, peaking at 1–3 h before decreasing; however, the extent of these expression-level changes varied among NaCl concentrations. The expression level of *BobOSR1* remained consistently low in cauliflower leaves across all treatments, indicating that it likely makes a negligible contribution to stress tolerance and hormone responses in cauliflower. For all stress treatments, *BobARL2* consistently showed the highest expression level and the most statistically significant changes in expression across experimental conditions, indicating that it may be a key stress-responsive gene in the *ARGOSs* in cauliflower.

### 2.7. Regulatory Effects of BobARL2 on Arabidopsis Root Growth Under ACC and NaCl Stress Conditions

**Figure 7:**
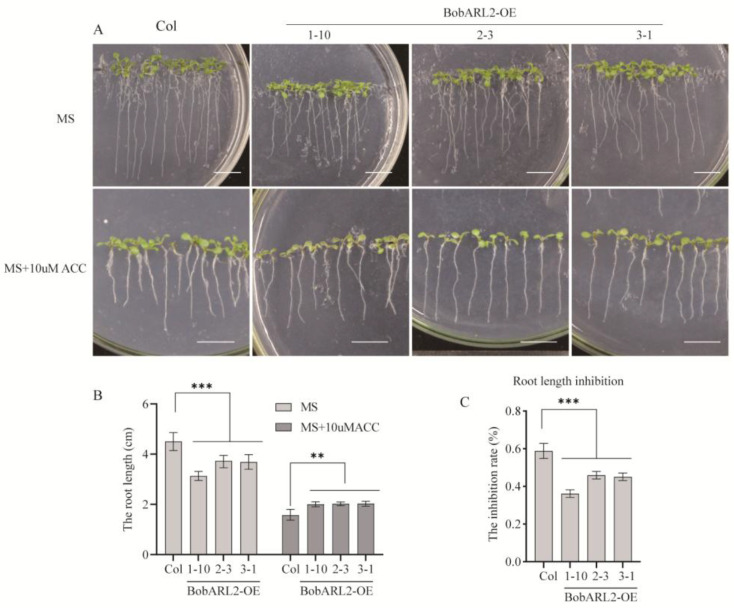
Phenotypic analysis of wild-type (Col) and BobARL2-OE transgenic Arabidopsis lines under normal conditions and ACC stress. (**A**) Representative images of seedling root growth 9 days after vertical placement on 1/2 MS medium with or without ACC. Scale bar = 1 cm. (**B**) Quantification of primary root lengths corresponding to (**A**). (**C**) Root growth inhibition rate. Data are presented as mean ± SD (n = 30). Significant differences were determined by two-way ANOVA followed by Tukey's multiple comparisons test (** *p* < 0.01, *** *p* < 0.001).

**Figure 8:**
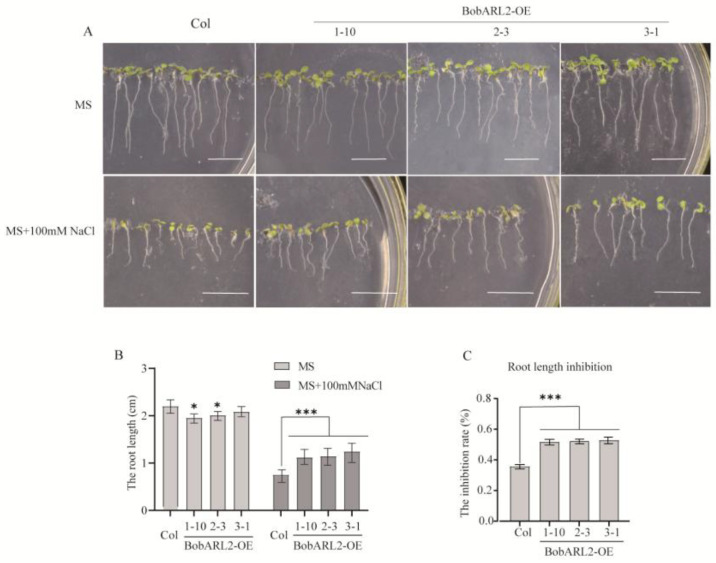
Phenotypic analysis of wild-type (Col) and BobARL2-OE transgenic Arabidopsis lines under NaCl stress. (**A**) Root growth of Col and BobARL2-OE transgenic seedlings vertically grown on 1/2 MS medium with or without NaCl for 9 days after germination. Scale bar = 1 cm. (**B**) Main root lengths of Col and BobARL2-OE transgenic Arabidopsis lines measured 9 days after transfer to plates with NaCl. (**C**) Root growth inhibition rate. Data are presented as mean ± SD (n = 30). Significant differences were analyzed by two-way ANOVA followed by Tukey’s multiple comparisons test (* *p* < 0.05, *** *p* < 0.001).

*BobARGOS* gene expression levels were influenced by various stresses, with *BobARL2* expression significantly affected by ACC and NaCl treatments. To functionally characterize *BobARL2*, we used wild-type Arabidopsis (Columbia) plants and T_3_ transgenic Arabidopsis lines in which *BobARL2* was expressed under the control of the 35S promoter to investigate the effects of ACC and NaCl stresses on root growth. When seedlings were grown vertically on Murashige and Skoog (MS) medium, the inhibitory effects of 10 μM ACC on root growth were significantly greater for the wild-type control than for *BobARL2*-overexpressing (*BobARL2*-OE) Arabidopsis lines (Figure 7). Furthermore, under NaCl stress conditions, *BobARL2*-OE transgenic plants had significantly longer roots than wild-type plants, demonstrating enhanced salt tolerance (Figure 8). These phenotypic observations imply that *BobARL2* overexpression decreases ethylene sensitivity in Arabidopsis.

### 2.8. Interaction Between BobARL2 and BobRTL4

**Figure 9:**
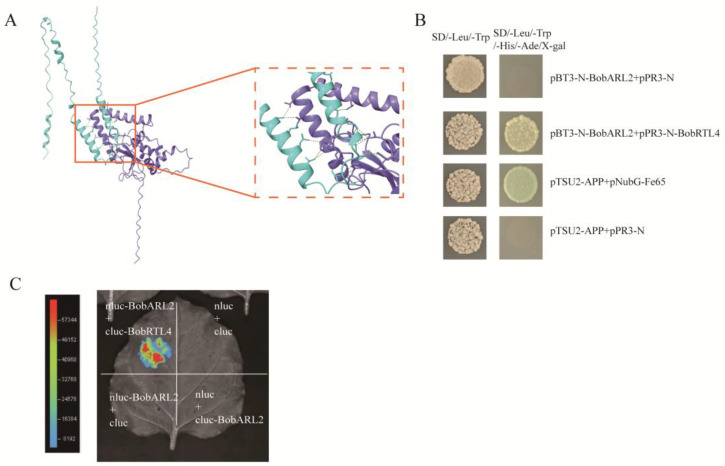
The interaction of BobARL2 and BobRTL4. (**A**) Molecular docking model of the protein complex (BobARL2, blue; BobRTL4, purple) predicted by AlphaFold3 and visualized with PyMol 2.6. The interaction was confirmed by Y2H (**B**) and LCI assays (**C**).

Previous studies have indicated that *BobARL2* is involved in ethylene signal transduction, and several *ARGOS* gene family members encode proteins that can interact with key components of the ethylene signaling pathway, including RTE1 and RTL4 [12]. Accordingly, we used AlphaFold3 to predict the three-dimensional structure of the BobARL2–BobRTL4 complex and characterize the interaction between the two proteins. The predicted structural model, which was visualized using PyMOL (Figure 9A), reveals the interaction between the two proteins. To experimentally validate this prediction, we conducted a yeast two-hybrid assay (Y2H) (Figure 9B) and luciferase complementation imaging (LCI) assay (Figure 9C). The results confirmed that BobARL2 directly interacts with BobRTL4, suggesting that BobARL2 may modulate ethylene signaling through this molecular association.

## 3. Discussion

In plants, ARGOS is a type of hormone-responsive protein associated with organ enlargement and stress responses [12,15,16,19,22,23]. *ARGOS* genes encode transmembrane proteins with an OSR domain [10,15,22]. Because of their key roles and the increasing availability of genomic data, researchers have identified multiple *ARGOS* genes in many species, including wheat, maize, and tobacco [14,19,23]. However, there has been no systematic identification and analysis of *ARGOS* genes in Brassicaceae, which includes many important vegetable crops. Therefore, in this study, *ARGOS* genes were identified in several recently published genomes of Brassicaceae species cultivated as vegetable crops (*B. oleracea*, *B. rapa*, *R. sativus*, and *B. nigra*) and then analyzed in terms of their structures, physicochemical properties, evolutionary characteristics, and promoter cis-acting elements as well as their responses to various stresses in cauliflower. Our findings suggest that *ARGOS* genes have moderately similar sequences and functions, but they vary regarding their tissue-specific roles and responses to diverse stresses. Therefore, these findings offer valuable insights into *ARGOS* genes and serve as a resource for future studies aimed at their functional characterization.

Phylogenetic analysis is a powerful tool for inferring the evolutionary history of genes across species [30]. On the basis of phylogenetic and evolutionary analyses, *ARGOS* genes were classified into three distinct clades (Figure 1). All *ARGOS* and *ARL* genes from different species were clustered together (Cluster III), suggesting that they are highly conserved in terms of sequence and function, which is consistent with the findings of previous studies [15,19,22]. Compared to those from other species, the *ARL* genes from the three *B. oleracea* varieties exhibited a closer evolutionary relationship among themselves. This indicates that they originated from a relatively recent gene duplication event specific to *B. oleracea* and have since undergone minimal functional divergence, maintaining a high degree of sequence and functional conservation among themselves. The expansion and diversification of the *ARGOS* genes are likely the consequences of the whole-genome triplication event shared by the ancestral Brassica species [31]. Following this triplication, ancestral *Brassica* species underwent diploidization involving extensive gene loss and functional diversification of the retained genes [31]. This process led to the elimination of a substantial number of genes. The extent of gene loss within the *ARGOS* genes following polyploidization varies across species. Notably, all three examined *B. oleracea* genomes lack an *OSR1* homolog, suggesting that the loss of this gene likely occurred prior to the divergence of the *B. oleracea* lineage. This implies that one or more genes with similar or identical functions may have gradually compensated for the absence of *OSR1* during the evolution of Brassicaceae species grown as vegetable crops.

Identifying cis-acting elements in promoter regions represents a fundamental strategy for studying temporospatial gene expression patterns, as well as gene expression related to tissue growth and developmental processes [32]. These cis-regulatory elements are essential for transcriptional regulation because they serve as binding sites for specific transcription factors [32,33]. In this study, an analysis of *ARGOS* promoter regions revealed numerous stress response-related cis-regulatory elements (Figure 3). Light-responsive elements were the predominant motifs among the *ARGOS* genes, with Box-4—which is a key regulatory element involved in light signaling—being present in all members. The promoter regions of most *ARGOS* members also contain other light-responsive elements such as GT1-motif and G-box, which are associated with the regulation of photoperiod and circadian rhythm [34]. Additionally, cis-acting elements such as ABRE and ERE were abundantly identified. The ABRE motif, which serves as the binding site for ABA-responsive element binding proteins (AREB/ABFs), plays a crucial role in ABA-mediated stress response signaling [35,36]. Moreover, ethylene transcription factors mediate ethylene signal transduction by binding to the ERE motif, thereby regulating the expression of a series of genes involved in plant growth, development, and stress response [37,38]. Collectively, these results demonstrate the critical functions of *ARGOSs* in mediating responses to photoperiod, hormonal cues, low temperature, and drought stress. 

Subcellular localization results indicated that BobARGOS proteins (except BobOSR1) are predominantly localized to the endoplasmic reticulum (Figure 4), which is consistent with the result of a previous study [10]. Notably, earlier research on Arabidopsis showed that the endoplasmic reticulum also harbors ethylene receptors and other early signaling components of the ethylene pathway [16]. This may reflect spatial coordination of ethylene-related signaling molecules.

Previous studies showed that homologous genes across different species often retain similar functions throughout evolution [2,3,4]; the tissue-specific expression patterns of these genes are closely linked to their functions [2,3,4]. *AtARGOS* genes exhibit distinct tissue- and organ-specific expression patterns during Arabidopsis development. For example, *OSR2* is most highly expressed in expanding leaves, inflorescences, and flowers [11], whereas *ARL* expression levels are highest in cotyledons, roots, expanding leaves, flowers, and siliques [22]. *ARGOS* is predominantly expressed in flowers, young siliques, and roots [15]. Similarly, in tobacco, *NtARL1* is most highly expressed in flowers and the stem [19]. In wheat, *TaARGOS* expression levels are highest in the first true leaves, roots, and flowers [23]. These expression patterns are partially consistent with the observed expression of *BobARGOS* genes in different cauliflower tissues. Notably, *BobARL1* and *BobARL2* are significantly up-regulated in leaves, with *BobARL2* also highly expressed in expanding leaves, roots, and siliques. Interestingly, *BobARL2* is also expressed at high levels in the curd, suggesting a potential role in curd development (Figure 5C). *BobARGOS* expression levels in cauliflower leaves vary with leaf age, with *BobARL2* expression peaking in the third leaf (from the outermost to innermost leaves) (Figure 5A). This expression peak in a maturing leaf, rather than in the innermost youngest leaf where cell division is most active, suggests that *BobARL2* may be more involved in the phase of cell expansion. This observation is consistent with the documented role of its Arabidopsis homolog, *ARL*, which primarily promotes cell expansion rather than division [22]. It should be noted that this is a correlative observation, and direct evidence from cellular measurements is required to confirm this hypothesis. Moreover, during curd development, *BobARL1*, *BobARL2*, and *BobARL3* expression patterns vary substantially, suggesting that the proteins encoded by these genes have diverse functions in cauliflower (Figure 5). Considering these genes encode small transmembrane proteins with an OSR domain, their high expression levels in specific tissues imply they may be involved in the development of these tissues.

*ARGOS* gene expression is induced by multiple stresses and varies among species, suggesting that these genes play diverse roles in stress resistance. For example, *AtARGOS* expression is significantly induced by low-temperature stress (8 °C) and high ethephon concentrations (e.g., 5 mM). The expression of *AtARL* in Arabidopsis is reportedly induced by NaCl and 5 mM ethephon [19]. In wheat, the expression of *TaARGOS-D* is induced by ABA, NaCl, and drought tolerance, while the expression levels of both *TaARGOS-A* and *TaARGOS-B* are upregulated under drought conditions [23,39]. In tobacco, *NtARL1* expression is induced by IAA and NAA. In the present study, five *BobARGOS* genes were differentially expressed in response to high- and low-temperature stresses, salt stress, and various hormones (Figure 6). Specifically, *BobARL2* expression was upregulated by ACC and NaCl at different concentrations, which is consistent with the observed effects of ACC and NaCl on *NtARL1* expression in tobacco [19]. In contrast to a previous study [22], the current study indicated *BobARGOS* expression levels were not significantly upregulated by BR. In cauliflower, the expression levels of *ARL* homologs (*BobARL1*, *BobARL2*, and *BobARL3*) increased under low-temperature stress conditions, whereas *BobARGOS* expression was not induced, which is in contrast to the reported expression-level changes to homologous genes in tobacco [19].

Plants within the Brassicaceae family are highly susceptible to various abiotic stresses during growth. Recent studies have revealed that enhancing stress tolerance in Brassica vegetables can be achieved by modulating the expression of key genes. For instance, upregulation of Plasma membrane intrinsic protein 2-1(PIP2-1) expression in radish has been shown to significantly improve salt tolerance [40]. Moreover, protein phosphatase (BrPP5.2) has been demonstrated to regulate heat-responsive gene expression through its intrinsic chaperone activity in *B. rapa* [41]. Similarly, phytohormones also play crucial roles in enhancing stress tolerance in Brassica vegetables. In radish, *C-repeat Binding Factor 2* (*RsCBF2*) and *C-repeat Binding Factor 2* (*RsERF18*) improve salt tolerance via the ethylene synthesis pathway [40], while in Brassica napus, BnSIP1-1 contributes to salt and osmotic stress resistance by modulating ABA biosynthesis and signaling [42]. 

Numerous studies showed that the proteins encoded by *ARGOS* genes play critical roles in organ development and stress tolerance, primarily by modulating ethylene signaling [13,14,16,19,21,22]. These findings suggest that *ARGOS* gene members may be involved in a negative feedback mechanism that decreases plant sensitivity to ethylene, enabling plants to respond to a relatively broad range of ethylene concentrations. Proteins such as RTE1, its homolog RTL4, and Responsive-to-antagonist1 (RTA1) are involved in ethylene perception modulation [43,44,45]. Both RTE1 and RTL4 enhance the activity of Ethylene response1 (ETR1), thereby negatively regulating ethylene signal transduction [44,45,46,47,48]. In maize, ARGOS proteins interact with RTE1 and RTL4, collectively regulating ethylene signal perception and early transduction, which ultimately enhances abiotic stress tolerance and improves crop yield [13,14,47,48]. 

In this study, we demonstrated that overexpression of *BobARL2*, an *ARGOS* homolog from cauliflower, reduced ethylene sensitivity in transgenic Arabidopsis, as evidenced by root elongation assays under ACC treatment (Figure 7), which is consistent with earlier findings on *ARGOS* family genes. AlphaFold3 is an AI-based software for predicting protein structures, which has been increasingly applied to predict protein-protein interactions in plants due to its high accuracy and flexibility [49]. For example, AlphaFold3 has been used to analyze the interaction characteristics of *GLABROUS1* enhancer-binding protein (GeBP3) and GeBP5 under drought and other stresses in pepper [50]. In this study, we employed AlphaFold3 to predict the interaction between BobARL2 and BobRTL4, and the results suggest the presence of multiple potential interaction sites between the two proteins. The interaction between BobARL2 and BobRTL4 was further verified through yeast two-hybrid experiment and LCI assay, which may contribute to the decreased ethylene sensitivity and increased salt tolerance observed in transgenic Arabidopsis plants expressing *BobARL2*.

However, the precise molecular mechanism by which the BobARL2-BobRTL4 complex regulates ethylene transduction, such as through affecting the stability or activity of ethylene receptors or downstream signaling elements, remains an important subject for future investigation. Moreover, plant stress responses are often mediated by hormonal crosstalk rather than isolated pathways. For instance, auxin and ethylene cooperatively regulate root development and architecture, a key adaptive mechanism for drought and salinity tolerance [51], and ethylene can compensate for ABA deficiency in stomatal closure, enhancing drought tolerance in tomato [52]. Therefore, Therefore, *BobARL2* may function as a node within a broader hormone network, possibly integrating signals from ethylene, ABA, and auxin—a hypothesis that merits further investigation. 

While this study provides functional insights into BobARL2, it primarily relied on root elongation as a phenotypic readout. Future studies should include additional parameters such as shoot biomass and ion content to fully elucidate its mechanistic role. It is also important to note the limitations of using Arabidopsis as a heterologous system. Given the potential role of BobARL2 in the development of the unique cauliflower curd, future work should prioritize genetic validation in cauliflower itself to unequivocally define its functions in both stress adaptation and curd development.

## 4. Materials and Methods

### 4.1. Sequence Acquisition and Genome-Wide Identification of ARGOS Genes in Different Species

Whole-genome sequences from three *B. oleracea* subspecies (TO1000 kale-like rapid cycling morphotype, HDEM broccoli, and Korso cauliflower) were analyzed. The TO1000 genome was sourced from EnsemblPlants (http://plants.ensembl.org/, accessed on 19 March 2025) [25], whereas Korso and HDEM genomes were obtained from Figshare (https://figshare.com/collections/Korso_and_OX_heart_genome_assemblies_and_annotations/5392466/2, accessed on 19 March 2025) [6] and Genoscope (http://www.genoscope.cns.fr/, accessed on 19 March 2025) [29], respectively. Additional genome sequences for *B. rapa* ssp. Chiifu, *B. nigra* YZ12151, and *R. sativus* XYB-36-2 were retrieved from the BRAD database (http://brassicadb.org/brad/, accessed on 19 March 2025) [26,27,28]. The sequences of four Arabidopsis ARGOS proteins were obtained from the TAIR database (https://www.arabidopsis.org/, accessed on 19 March 2025) and used as queries for local BlastP searches (E-value = 10^−5^) of the genomes of the selected species. This approach identified candidate ARGOS sequences. To confirm homology, these candidates were further analyzed via BlastP searches of the TAIR Arabidopsis protein database. The top hits were examined to confirm that they correspond to Arabidopsis *ARGOS* genes, leading to the successful identification of *ARGOS* genes in seven species.

Physicochemical properties of ARGOS proteins (e.g., molecular weight and theoretical isoelectric point) were determined using the ExPASy 5.0 online tool (http://expasy.org) [53]. TMHMM Server v2.0 was used to predict ARGOS transmembrane structures (https://services.healthtech.dtu.dk/services/TMHMM-2.0/, accessed on 19 March 2024), whereas CELLO v.2.5 was used to predict subcellular localizations (http://cello.life.nctu.edu.tw/, accessed on 19 March 2024) [54].

### 4.2. Sequence Alignment and Phylogenetic Analyses of ARGOS Genes

We used the global alignment tool ‘Needle’ from the EMBOSS 6.6.0 suite to calculate ARGOS protein sequence identities and similarities [55]. Whole ARGOS amino acid sequences were aligned using the MUSCLE 3.8.31 program [56]. The protein sequences were aligned using MultAlin software (version 5.4) (http://multalin.toulouse.inra.fr/multalin/ accessed on 20 March 2024) [57]. Phylogenetic trees were constructed using MEGA 7.0 and the neighbor-joining method based on the Jones–Taylor–Thornton model, with 1,000 bootstrap replicates to assess the statistical support of each node [58]. We applied uniform rates, homogeneous lineages, and excluded gaps with a site coverage cutoff of 70%. An additional phylogenetic tree was constructed for ARGOS proteins using the same method. 

### 4.3. Synteny Analysis of OSR1 in Brassica oleracea and Arabidopsis

To investigate the genomic basis for the absence of the *OSR1* in *Brassica oleracea*, a comparative synteny analysis was performed. The genomic region harboring *AtOSR1* in *Arabidopsis thaliana* was used as a query. Alignments were conducted using SynOrths (http://brassicadb.cn/#/RegionMicroSynteny/ accessed on 21 March 2024) [59].

### 4.4. Analyses of ARGOS Gene Structures and Conserved Motifs

On the basis of ARGOS amino acid sequences, we analyzed conserved motifs using MEME Suite v5.5.2 (https://meme-suite.org/, accessed on 20 March 2024). TBtools was used to analyze and visualize conserved domains in ARGOS proteins [60]. Furthermore, gene structures were visualized using GSDS2.0 (http://gsds.gao-lab.org/, accessed on 20 March 2024) [61].

### 4.5. Analysis of ARGOS Promoter Cis-Acting Elements

The 2,000 bp sequence upstream of the start codon was extracted and analyzed as the promoter region of *ARGOS* genes. Cis-acting elements in the promoters of ARGOS genes were predicted with PlantCARE (http://bioinformatics.psb.ugent.be/webtools/plantcare/html/, accessed on 20 March 2025) [62] and visualized using TBtools (v2.331).

### 4.6. Plant Materials and Treatments

Korso cauliflower seeds were germinated at 24 °C for 2 days in darkness. The resulting seedlings were grown in plastic pots filled with soil under controlled conditions in a greenhouse [16-h light (24 °C)/8-h dark (20 °C)]. To investigate *BobARGOS* expression patterns in various cauliflower tissues and curd development stages, we used published transcriptome data [6] for different Korso cauliflower tissues (root, stem, leaf, flower, and curd) and various shoot apex/curd developmental stages (vegetative growth, transformation, enlargement, maturation, and bolting). To further analyze *BobARGOS* expression in different developmental stages, we selected 6-week-old cauliflower seedlings with seven leaves and sequentially sampled the first to seventh leaves (L1–L7 from the outermost to innermost leaves).

To explore *BobARGOS* expression profiles in response to abiotic stresses, 6-week-old cauliflower seedlings were treated with various hormones: 50 μM 6-BA, 5 μM IAA, 10 μM NAA, 1 μM EBL, 5 μM GA3, 10 μM abscisic acid, 0.5 mM ACC, and 2 mM 1-MCP as well as NaCl at different concentrations (90, 150, and 380 mM). The third leaf of each plant was collected at 0.5, 1, 3, and 5 h post-treatment. To examine *BobARGOS* expression under temperature stress conditions, 6-week-old cauliflower seedlings were incubated at 8 °C (low-temperature stress) or 42 °C (high-temperature stress). The third leaf was collected at 0.5, 1, 3, and 5 h post-treatment.

For the control group, 6-week-old cauliflower seedlings were sprayed with water and the third leaf was collected. All collected samples were rapidly frozen in liquid nitrogen and stored at −80 °C for the subsequent analysis of *BobARGOS* expression in leaves at different development stages or in response to different abiotic stress conditions.

### 4.7. qRT-PCR Analysis

Total RNA was extracted from cauliflower using a FastPure Plant Total RNA Isolation Kit (Vazyme, Nanjing, China), after which cDNA was synthesized using a PrimeScript™ RT Reagent Kit and gDNA Eraser (TaKaRa, Dalian, China). Primers for qRT-PCR were designed using Primer Premier 5.0 software (Table S4). The cauliflower *Actin* gene was selected as the reference control, based on its demonstrated expression stability in cauliflower under similar abiotic stress and hormone treatment conditions [63]. TB Green® Premix Ex Taq™ (TaKaRa, Dalian, China) and the following program were used to conduct qRT-PCR analyses: 95 °C for 30 s; 40 cycles of 95 °C for 10 s, 60 °C for 30 s, and 72 °C for 20 s. Relative gene expression levels were calculated using the 2−ΔΔCT method, with three technical replicates for each sample [64].

### 4.8. Subcellular Localization

Following the method of Dang et al. (2014), we determined the subcellular localization of BobARGOS proteins by transiently expressing eGFP fusions in *N. benthamiana* leaves via the pFGC system. Four *BobARGOS* genes (excluding the non-expressed *OSR1*) were cloned into the vector and introduced into *A. tumefaciens* EHA105. EHA105 cells were transformed with the constructed recombinant vectors or an empty pFGC vector (control). Cultures of the transformed *A. tumefaciens* cells were adjusted for an OD_600_ of 0.8, then infiltrated into tobacco leaves as previously described [65]. For co-expression assays, cultures containing the eGFP-BobARGOS construct, the endoplasmic reticulum marker RFP-HDEL, and the gene silencing suppressor p19 were mixed in a 1:1:1 ratio. Each treatment consisted of three biological replicates, with each replicate comprising three plants. Confocal imaging was performed 48 hours post-infiltration using an LSM780 microscope (Carl Zeiss), with parameters optimized for each fluorophore. Sequences of the primers used for cloning *BobARGOS* genes are provided in Table S4.

### 4.9. Vector Construction and Plant Transformation

The full-length *BobARL2* coding sequence was amplified by PCR using gene-specific primers (upstream primer: 5′-cgctctagaactagtggatccATGATTCGTGAAATCTCCGGTC-3′; downstream primer: 5′-cttgatatcgaattcctgcagTTACATATAAGTTCTTGTTACATGTTTGGC-3′; underlined sequences indicate restriction enzyme cut sites). The p35S::BobARL2 construct was obtained by inserting *BobARL2* into the pYBA1302 vector for the subsequent expression under the control of the CaMV 35S promoter and NOS terminator. Transgenic Arabidopsis plants were generated via floral dip transformation. We collected two leaves from each transgenic and control plant and then extracted genomic DNA using the CTAB (hexadecyltrimethylammonium bromide) method for PCR analyses. Homozygous *BobARL2*-OE transgenic seeds (T_3_ generation) were used for the functional characterization of *BobARL2*.

### 4.10. ACC and NaCl Treatments of Transgenic Arabidopsis

Wild-type Arabidopsis and homozygous transgenic seeds (T_3_ generation) were stratified at 4 °C for 2 days to promote germination. The resulting seedlings were grown on half-strength MS medium under controlled conditions (22 °C, 16-h light/8-h dark cycle, and 60% relative humidity). To examine the effects of ACC and NaCl on seedling growth, 3-day-old uniformly growing seedlings were transferred to MS medium supplemented with 10 μM ACC or 100 mM NaCl. Root length was measured at 9 days post-ACC treatment and at 6 days post-NaCl treatment. Each treatment included three biological replicates, with each replicate consisting of 10 plants.

### 4.11. Predicting the Interaction Between BobARL2 and BobRTL4

The structural model of the BobARL2-BobRTL4 complex was generated with AlphaFold3 (https://golgi.sandbox.google.com/; accessed on 2 March 2024), and the binding sites visualized using PyMol 2.6 (Schrödinger, LLC, New York, NY, USA).

### 4.12. Protein Interaction Analysis

For the yeast two-hybrid assay, the coding sequences of *BobARL2* were cloned into the pBT3-N plasmid to construct the prey vector, whereas the *BobRTL4* coding sequence was inserted into the pPR3-N plasmid to generate the bait vector. Both the bait and prey plasmids were co-transformed into Y2HGold yeast strains. The pair of plasmids pTSU2-APP and pNubG-Fe65 was used as a positive control. The pair of plasmids pTSU2-APP and pPR3-N was used as a negative control. Transformed yeast cells were first cultured on SD medium lacking leucine and tryptophan (SD/–Leu/–Trp) for two days. Subsequently, the yeast colonies were transferred onto SD medium deficient in leucine, tryptophan, histidine, and adenine (SD/–Leu/–Trp/–His/–Ade) to assess protein–protein interactions.

To validate the protein-protein interaction, constructs encoding BobARL2-nLuc, BobRTL4-cLuc, BobARL2-cLuc, along with their corresponding empty vectors, were individually transformed into *A. tumefaciens* strain GV3101. Equal volumes of differentially combined bacterial suspensions were co-infiltrated into leaves of *N. benthamiana*. Luminescence signals were detected using a chemiluminescence imaging system (Clinx 6100, China) following application of D-Luciferin potassium salt (40901ES01, Yeasen, China). 

## 5. Conclusions

This study presents a genome-wide identification and functional analysis of *ARGOS* genes in three *B. oleracea* varieties (kale-like rapid cycling morphotype, cauliflower, broccoli), along with *B. rapa*, *B. nigra*, and *R. sativus*. The characterization included analyses of phylogenetic relationships, chromosomal locations, gene structures, protein motifs, evolutionary patterns, promoter cis-elements, and expression profiles across various tissues and under abiotic stresses. Our results reveal that *ARGOS* genes exhibit both evolutionary conservation and diversity among Brassica species and play important roles in abiotic stress and hormonal responses in cauliflower. In particular, *BobARL2* was shown to contribute significantly to stress adaptation, underscoring its functional importance and warranting further mechanistic investigation. These findings provide a foundation for future functional studies on *ARGOS* genes in *B. oleracea* and related species.

## Data Availability

All data are included in the article. RNA-seq raw data are available at the NCBI BioProject database under accession number PRJNA546441.

## Supplementary Materials

The following supporting information can be downloaded at: https://www.mdpi.com/article/10.3390/ijms26199810/s1.

## Abbreviations

The following abbreviations are used in this manuscript:

ARGOS	Auxin-Regulated Gene Involved in Organ Size
BobRTL4	Reversion-to-ethylene sensitivity Like4
OSR	Organ size-related
ANT	Aintegumenta
BRI1	Br insensitive 1
RTE1	Reversion-to-ethylene sensitivity1
qRT-PCR	quantitative real-time polymerase chain reaction
IAA	including indole-3-acetic acid
NAA	1-naphthaleneacetic acid
6-BA	6-benzylaminopurine
1-MCP	1-methylcyclopropene
GA3	gibberellin
ACC	1-aminocyclopropane-1-carboxylic acid
EBL	24-epibrassinolide
RTA1	Responsive-to-antagonist1
ETR1	Ethylene response1
WAT1	Walls Are Thin1
LCI	luciferase complementation imaging
Cyc D3;1	Cyclin D3;1
RsCBF2	C-repeat Binding Factor 2
RsERF18	C-repeat Binding Factor 2
MS	Murashige and Skoog
Y2H	yeast two-hybrid assay
GeBP3	GLABROUS1 enhancer-binding protein
