# Supplementary figures and images for: ARGOS Genes in Cauliflower: Genome-Wide Identification and Functional Validation of BobARL2 Under Abiotic Stresses
Source: Int J Mol Sci. 2025 Oct 9;26(19):9810. doi: 10.3390/ijms26199810 (PMC12524420; doi:10.3390/ijms26199810)

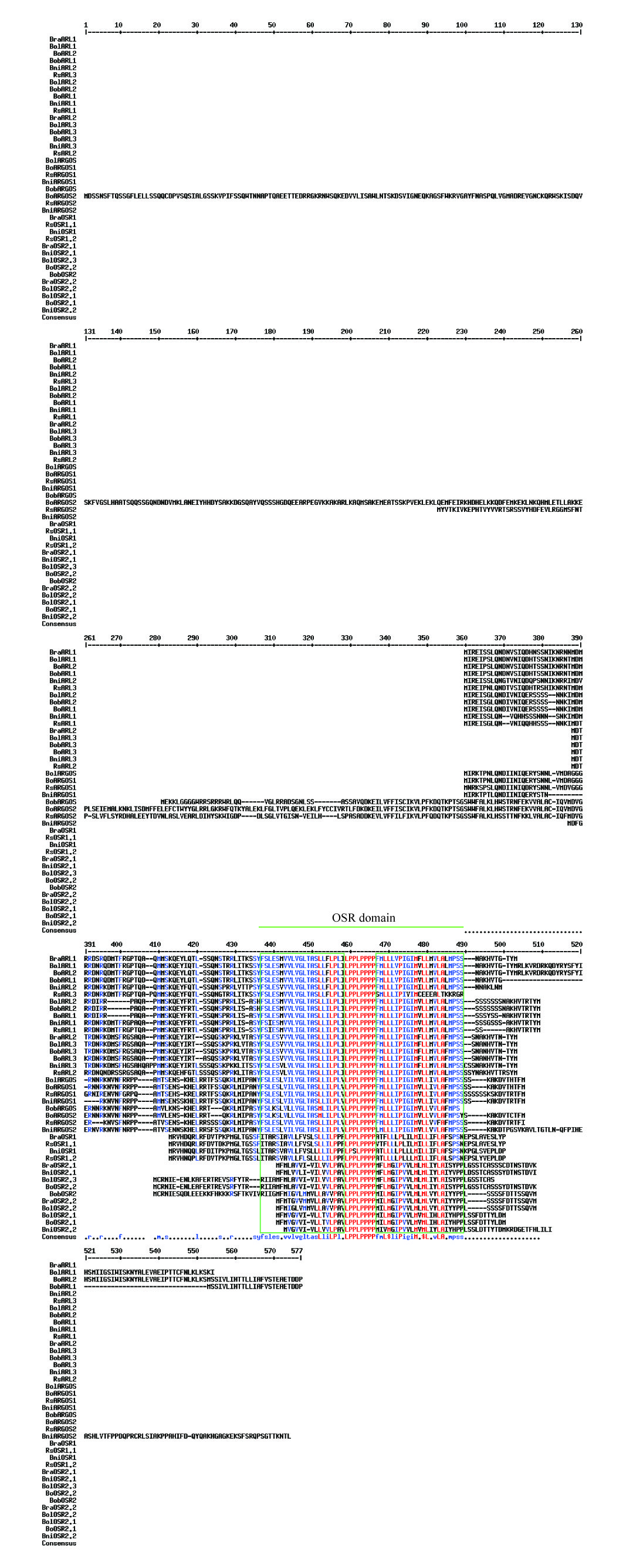

Supplement: Supplementary file 1 [file ijms-26-09810-s001.zip › Figure S1.jpg]
